# Wide variation in surgical techniques to repair incisional hernias: a survey of practice patterns among general surgeons

**DOI:** 10.1186/s12893-021-01261-9

**Published:** 2021-05-24

**Authors:** Simon MacDonald, Paul M. Johnson

**Affiliations:** 1 Division of General Surgery, Dalhousie University, Halifax, NS Canada; 2 Department of Community Health and Epidemiology, Dalhousie University, Halifax, NS Canada; 3 QEII Health Sciences Centre, Room 806 Victoria Building, VGH Site, 1276 South Park St., Halifax, NS B3H 2Y9 Canada

**Keywords:** Surgery, Incisional hernia, Mesh, Practice patterns

## Abstract

**Background:**

The purpose of this research was to examine the self-reported practice patterns of Canadian general surgeons regarding the elective repair of incisional hernias.

**Methods:**

A mail survey was sent to all general surgeons in Canada. Data were collected regarding surgeon training, years in practice, practice setting and management of incisional hernias. Surgeons were asked to describe their usual surgical approach for a patient with a midline incisional hernia and a 10 × 6 cm fascial defect.

**Results:**

Of the 1876 surveys mailed out 555 (30%) were returned and 483 surgeons indicated that they perform incisional hernia repair. The majority (62%) have been in practice > 10 years and 73% regularly repair incisional hernias. In response to the clinical scenario of a patient with an incisional hernia, 74% indicated that they would perform an open repair and 18% would perform a laparoscopic repair. Ninety eight percent of surgeons would use mesh, 73% would perform primary fascial closure and 47% would perform a component separation. The most common locations for mesh placement were intraperitoneal (46%) and retrorectus/preperitoneal (48%). The most common repair, which was reported by 37% of surgeons, was an open operation, with mesh, with primary fascial closure and a component separation.

**Conclusions:**

While almost all surgeons who perform incisional hernia repairs would use permanent mesh, there was substantial variation reported in surgical approach, mesh location, fascial closure and use of component separation techniques. It is unclear how this variability may impact healthcare resources and patient outcomes.

**Supplementary Information:**

The online version contains supplementary material available at 10.1186/s12893-021-01261-9.

## Background

Incisional hernias are one of the most common problems seen in general surgery practice. While expectant management is appropriate for some patients, many patients with an incisional hernia will require surgery [1]. Unfortunately, recurrence after repair is common with some studies reporting rates of more than 50% among patients who have been followed for at least 10 years after surgery [2]. To some extent, this likely reflects the fact that there is very little high-quality evidence to guide management and it is often unclear which approaches, if any, are associated with the best outcomes. In contrast to other surgical problems, there are many different techniques and materials that can be used to repair an incisional hernia. Surgeon decision-making is further complicated by the frequent introduction of new materials and novel procedures for managing these patients. This is illustrated by the fact that in just 2 years (2013–2015) 77 new hernia meshes were approved for clinical use by the Food and Drug Administration in the United States [3]. Given the variety of mesh products currently available and different surgical approaches described in the literature, the potential for variation in clinical practice and confusion among surgeons is likely as high as it has ever been. However, very little is known about the current management of incisional hernias among practicing surgeons or how much variation may exist. Therefore, the purpose of this research was to describe the self-reported practice patterns of Canadian general surgeons for the elective management of incisional hernias.

## Methods

A mail survey was sent to all general surgeons in Canada between. Mailing addresses were obtained from the Canadian Medical Directory. A cover letter explaining the purpose of the study was included along with the survey. Each respondent was provided with a stamped, addressed envelope to return the questionnaire. The survey was translated into French, and both French and English versions were sent to surgeons in Quebec to complete in the language of their choice. Only one survey was sent to each surgeon and there were no reminders or follow-up surveys.

The survey was designed in conjunction with experts in ventral hernia repair from our institution (Additional file 1: Appendix A). It was then reviewed by academic and community general surgeons for content and clarity and changes were made based on the feedback. The survey collected data regarding years in practice, practice setting, surgical training, frequency of hernia repair, and whether or not surgeons considered themselves hernia repair “experts”. Surgeons were asked to describe their usual surgical technique for a patient with a midline incisional hernia following a right hemicolectomy with a 10 × 6 cm fascial defect. This included surgical approach, use of mesh, type of mesh, mesh placement, mesh fixation technique, fascial closure and use of component separation. The following definitions were used for mesh placement; onlay: above the fascia, inlay: retrorectus, preperitoneal (within the abdominal wall) and sublay; intraperitoneal.

Descriptive statistics were calculated using Microsoft Excel. Categorical variables were described using proportions and frequencies and chi square analysis was used for comparison between groups. Responses were compared between self-reported experts and non-experts. No information was available for the non-responders.

Approval for this study was obtained from the Nova Scotia Health Authority research ethics board. All methods were carried out in accordance with relevant guidelines and regulations. By filling out and returning the survey the participants provided informed consent for involvement with the study.

## Results

Of the 1837 surveys mailed out to general surgeons across Canada 555 (30.0%) were returned. Twenty-seven respondents indicated that were no longer in practice and 45 reported that they did not perform incisional hernia repairs. The remaining 483 respondents comprised the study cohort (Table 1). Among these respondents, 76% regularly perform incisional hernia repairs, and 37% of those considered themselves experts. In contrast only 1.5% and 0.2% of surgeons who repair incisional hernias infrequently or only when on call consider themselves experts, respectively.

**Table 1:** Self-reported demographic and practice characteristics of 483 Canadian general surgeons who perform incisional hernia repairs

Years in practice	
< 5	16%
5–10	22%
10–20	30%
> 20	32%
Practice setting	
Academic teaching hospital	32%
Community hospital serving > 500,000	15%
Community hospital serving 100,000–500,000	25%
Community hospital serving < 100,000	28%
Fellowship trained	
Yes	52%
No	48%
Type of Fellowship	
Minimally invasive surgery	23%
Trauma/ICU	19%
Colorectal	16%
Surgical oncology	13%
Train general surgery residents	
Yes	66%
No	34%
Frequency of hernia repair	
Regularly	73%
Infrequently	23%
Only on call	4%

Given the clinical scenario of a patient with an incisional hernia following a right hemicolectomy with a 10 × 6 cm midline fascial defect, the majority of surgeons indicated that they would perform an open repair (74%), followed by laparoscopic (18.4%) and hybrid (7.6%) approaches. Almost all surgeons reported that they would use a permanent mesh to repair the hernia (Table 2) and 93% would place anchoring sutures. Seventy-three percent of surgeons reported that they would perform a primary fascial closure and just under half would perform a component separation (Table 3). Surgeons performing open procedures were more likely to use primary fascial closure compared to those who would perform laparoscopic operations (83% versus 23%, p < 0.05). Of those who perform a component separation 57% indicated that they would perform a posterior component separation. The self-reported experts were more likely to report using primary fascial closure (83% versus 67%, p < 0.05) and component separation (58% versus 39%, p < 0.05) compared to the non-experts.

**Table 2:** Self-reported use of mesh to repair an incisional hernia with a 10 × 6 cm midline fascial defect by Canadian general surgeons

Use mesh for repair	
Yes	98%
No	2%
Location of the mesh	
Sublay (intraperitoneal)	46%
Inlay (retrorectus & preperitoneal)	48%
Onlay (above the fascia)	6%
Type of mesh	
Permanent	95%
Synthetic absorbable	2.5%
Biologic	2.5%
Type of permanent mesh	
Light weight	47%
Medium weight	38%
Heavy weight	2%
I don’t know	13%

**Table 3:** Self-reported use of primary fascial closure and component separation to repair an incisional hernia with a 10 × 6 cm midline fascial defect by Canadian general surgeons

Perform primary fascial closure	
Yes	73%
No	27%
Perform a component separation	
Yes	49%
No	51%
Perform a transversus abdominus release (TAR)	
Yes	28%
No	68%
Surgeon unfamiliar with procedure	4%

The most common “overall surgical approach” was an open repair with mesh and primary fascial closure aided by a component separation. This was described by 37% of all respondents and 40% of the self-reported experts (Table 4). When only considering mesh location, primary fascial closure and component separation twelve different operations were described to repair the hernia in the clinical scenario (Table 5). The most common operation was an open repair, with an intraperitoneal mesh, with primary fascial closure and a component separation.

**Table 4:** The most common self-reported operative approaches for repair of an incisional hernia with a 10 × 6 cm midline fascial defect by Canadian general surgeons

Operative approach	Overall cohort (N = 483)	Self-reported experts (N = 180)	Self-reported non-experts (N = 303)
Open, with mesh, with primary fascial closure, with component separation	37%	40%	32%
Open, with mesh, with primary fascial closure, no component separation	19%	17%	22%
Laparoscopic, with mesh, no primary fascial closure, no component separation	13%	13%	14%
Open, with mesh, no primary fascial closure, no component separation	8%	2%	12%

Percentages do not total 100% as not all repairs are reported

**Table 5:** Self-reported location of mesh placement, use of primary fascial closure and component separation to repair an incisional hernia with a 10 × 6 cm fascial defect by 473 Canadian general surgeons

Mesh location			Primary fascial closure	Component separation	Surgeons performing procedure (%)
Inlay	Sublay	Onlay			
Yes	–	–	Yes	Yes	32.5
Yes	–	–	Yes	–	9.3
Yes	–	–	–	Yes	2.6
Yes	–	–	–	–	2.6
–	Yes	–	Yes	Yes	8.8
–	Yes	–	Yes	–	15.3
–	Yes	–	–	Yes	1.3
–	Yes	–	–	–	20.6
–	–	Yes	Yes	Yes	4
–	–	Yes	Yes	–	1.8
–	–	Yes	–	Yes	1
–	–	Yes	–	–	0.2

A “–” indicates that the technique was not used. Inlay = retrorectus & preperitoneal, Sublay = intraperitoneal, Onlay = on top of the fascia

## Discussion

Previously, very little has been known about the management of incisional hernias in Canada. In this study of self-reported practice patterns almost all surgeons reported that they would use permanent mesh. This is supported by the literature and is in keeping with current guidelines [1]. It was the most consistent practice among respondents. The use of laparoscopic surgery reported by surgeons in this study was considerably lower than that reported in previous clinical studies [4, 5]. This likely reflects the size of the defect described in our clinical scenario and the technical challenges associated with closing the fascia laparoscopically [6]. There was considerable variation among Canadian Surgeons regarding mesh type, mesh location, component separation techniques and the overall surgical approach they would use to repair an incisional hernia.

The variation in surgical practice observed in this study and in previous retrospective studies from Europe and the United States [4, 5, 7, 8] is likely due, in part, to a lack of high-quality evidence to guide care. Despite the fact that incisional hernia repair is a common procedure and is associated with high recurrence rates, very few randomized controlled trials (RCTs) have been performed to evaluate surgical techniques [9]. Most of the literature consists of observational studies and case series. Studies frequently combine heterogeneous patient populations with incisional, primary ventral and umbilical hernias [7]. Within studies focused exclusively on incisional hernias, there is often substantial variation regarding hernia size, hernia location and co-morbidities among patients [10, 11]. A recent systematic review of study quality among RCTs for incisional hernia repair highlighted several limitations. This included inconsistent techniques to determine hernia recurrence among studies (clinical exam and/or imaging) and variability in length of follow-up after surgery ranging from 1 to 64 months [9].

Another important limitation associated with the hernia literature is the lack of standardized definitions. This is of particular concern for the anatomic planes use for placement of mesh. The terms “onlay”, “overlay”, “inlay”, “underlay” and “sublay” have been used interchangeably to describe various repairs [12]. This has made hernia research, evaluation of outcomes and comparison of studies difficult. To address this issue Parker et al. have proposed a standardized classification of abdominal wall planes developed by a consensus panel of abdominal wall reconstruction experts [13]. They defined eleven potential locations for mesh placement, some of which have not commonly been described. Our survey was developed and sent out prior to this publication. The number of anatomic planes and definitions we used differed from those proposed by Parker et al. but were in keeping with what has been described in the literature. Given that the new standardized classification system was just published in 2020 it is too soon for it to have influenced the hernia repair literature. Whether or not it will gain widespread acceptance and how long this may take is unknown.

In addition to mesh placement, there are many other variables involved in repair of an incisional hernia including operative approach (open vs laparoscopic), type of mesh (permanent vs absorbable), fixation methods, mesh material, mesh weight, primary fascial closure and component separation. When considering all these possible factors, there are a remarkable number of different ways that an incisional hernia can be repaired. Ideally the choice of hernia repair technique should be tailored for each patient based on patient- and hernia-specific features. In the current study, Canadian surgeons reported 12 different ways they would repair the same hernia based on only three factors: mesh location, fascial closure and component separation. It is highly unlikely that each of these repairs will lead to the same outcomes, and differences in these techniques, and others, likely explain the variation in hernia recurrence rates among surgeons [7].

Previous studies have reported that patients treated by surgeons who have specialized training and clinical practice focused on abdominal wall reconstruction [14], or those who perform high volumes of incisional hernia surgery [15], have better outcomes compared to other patients. These improved results may be due to more appropriate choice of procedures and/or mesh materials, better surgical technique or a combination of both. In the present study, surgeons who considered themselves hernia repair experts were more likely to perform primary fascial closure in keeping with guidelines. However, there was still considerable variation in surgical procedures among the “experts”. While criteria to define expertise were not included in our study, such criteria have been described [16, 17]. Given the increasing complexity of incisional hernia surgery it has been suggested that there is a need for both specialized hernia centers and surgeons [16, 17]. Given the vast geography and population distribution of a country like Canada, centralization of hernia surgery in high volume tertiary-care centers is not realistic. However, limiting the number of surgeons in community and regional hospitals who perform incisional hernia surgery, and ensuring that they have appropriate training is potentially feasible. Given the variation in care reported in this study and ongoing evolution of incisional hernia repair techniques, continuing professional development events are needed now and in the future. This should include both hands-on courses and didactic teaching opportunities. Both have been shown to be important for improving the delivery of other surgical procedures in Canada [18].

Regardless of where incisional hernia surgery is performed or who performs it, a systematic approach to assessing new materials and surgical techniques is needed. The availability of numerous different types of hernia mesh, and the introduction of new materials, is facilitated by a system that does not require clinical trials to prove safety or effectiveness prior to use in clinical practice [3, 19]. All that is required is proof of “substantial equivalence” to materials and devices that have been previously approved. There is even less oversight regarding the introduction and adoption of new surgical techniques into clinical care. The use of component separation for incisional hernia repair is good example of this. Anterior component separation was first described 30 years ago while posterior component separation (TAR) was introduced in 2012 [20, 21]. A 2018 meta-analysis compared the impact of these two approaches on outcomes for patients with incisional hernias. The literature consisted of retrospective studies and case series that included a total of only 566 cases [22]. No RCTs or controlled prospective trials were identified. While short-term morbidity appears to be acceptable with these procedures [23], there are no data regarding the impact of anterior or posterior component separation on long-term respiratory function, pelvic stability, back pain, abdominal wall function or long-term hernia recurrence [1]. Despite this, the use of component separation appears to be increasing. It was used for 9% of open ventral hernia repairs from 2005 to 2013 in the United States [24], but in the present study 47% of survey respondents indicated that they would perform a component separation. Whether or not a component separation is appropriate for a patient with a 10 × 6 cm fascial defect is unclear. Given the lack of evidence to guide care surgeons may be unsure of when this procedure is clinically indicated. There may be other factors such a financial remuneration that influence the utilization of this procedure. We did not provide a definition of component separation with our survey. It is possible that surgeons who reported doing this procedure are in fact doing something other than what we had intended to capture.

There are several limitations associated with this research that should be considered. The response rate was 30%. It is possible that the non-responders were different from those who completed the survey leading to response bias. There are no data available for the non-responders. Despite the response rate, practice patterns for almost 500 surgeons from across the country were collected. Surgeons may have provided answers to the survey questions based on perceived best practices as opposed to what they would actually do in their own practice. However, there is evidence suggesting that self-reported physician practice patterns measured using a clinical vignette are similar to actual clinical practice [25]. This study was not linked to actual clinical care and it is unclear how the reported variation in hernia management impacts patient outcomes. The survey did not ask about the use of robotic repair as robotic surgery is not widely available in Canada. Finally, given the lack of standardized terms and definitions for certain elements of incisional hernia repair, some of the survey questions may have been unclear or confusing and the responses may not have been accurate.

## Conclusions

This study highlights that there is substantial variation in surgical techniques used to repair incisional hernias among Canadian General Surgeons. Although most surgeons reported that they would use permanent mesh with a primary fascial closure, more than 20% would perform a bridging repair and there was considerable variation among most other aspects of the procedure. There is a clear need for high quality research to help surgeons make rational decisions that optimize patient outcomes and use resources appropriately. Mesh location and the use of component separation techniques are important issues that should be considered in future clinical trials. The adoption of standardized nomenclature for abdominal wall planes will be critical to this. New materials and techniques for incisional hernia repair should not be introduced into clinical practice without mechanisms to evaluate their safety and effectiveness. Finally, there is a clear need to provide surgeons with continuing professional development opportunities now, given the current variation in care, and in the future as the evidence evolves for the management of incisional hernias.

## Supplementary Information

**Additional file 1: Appendix A.** Incisional hernia practice pattern survey.

## Data Availability

The datasets used and/or analysed during the current study are available from the corresponding author on reasonable request and with permission of our institutional research ethics board.
