# Supplementary material for: Wide variation in surgical techniques to repair incisional hernias: a survey of practice patterns among general surgeons
Source: BMC Surg. 2021 May 24;21:259. doi: 10.1186/s12893-021-01261-9 (PMC8145827; doi:10.1186/s12893-021-01261-9)
Supplement: Supplementary file 1 — Additional file 1: Appendix A. Incisional hernia practice pattern survey. [file 12893_2021_1261_MOESM1_ESM.docx]

**The management of incisional hernias: current practices of Canadian general surgeons.**

How long have you been in independent practice? <5 yrs 5-10 yrs >10-20 yrs >20 yrs

I am retired from clinical practice If so, thank you and please return the survey

What is your practice setting? Tertiary care academic teaching hospital

Community hospital population >500,000

Community hospital population 100,000-500,000

Community hospital population <100,000

Did you do a fellowship? Yes No

If Yes MIS Colorectal Breast Surgical oncology Hepatobiliary Endocrine Bariatric Other_________________

Do you perform incisional hernia surgery in your practice? Yes No

If “No” please stop and return the survey. Thank you.

If “Yes” select one I regularly perform incisional hernia surgery in my elective practice

I infrequently perform incisional hernia surgery in my elective practice

I only perform incisional hernia surgery when I’m on call

Do you train: Surgical residents Yes No

Surgical Fellows Yes No

Do you ever place mesh prophylactically (electively place mesh to prevent a hernia)? Yes No

If “yes” where do you use it? Incisional Parastomal Both

What type of mesh do you use? Permanent Synthetic absorbable Biologic

Clinical Scenario

You are seeing a 55 year old male who underwent an emergency laparotomy and right hemicolectomy for an obstructing colon cancer 5 years ago. This was complicated by a wound infection. He now has a symptomatic peri-umbilical incisional hernia. This is interfering with his quality of life and the decision has been to proceed with repair. He is otherwise healthy, takes no medications and is a non-smoker. His BMI is 28.

The hernia fascial defect is 10cm long and 6cm wide

Please describe your **usual** surgical management for this patient. Please answer all **6** questions

1. Operative approach (select one) Laparoscopic

Open

Hybrid (combination of laparoscopic and open)

1. Placement of a mesh Yes No
   1. If “yes” location of the mesh? (**select one**) Sublay (Intraperitoneal)

Inlay (retrorectus & preperitoneal)

Onlay (above the fascia)

If “yes” what type of mesh? (**select one**) Permanent

Synthetic absorbable

Biologic

If you selected “permanent mesh” is it? Light weight Medium weight

Heavy weight I don’t know

- 1. Do you place anchoring sutures for the mesh? Yes No
  2. How do you secure the mesh? Stapling/ tacking device Sutures Both I don’t
  3. What type of tacks/staples do you use? Absorbable Permanent N/A

1. Do you perform a component separation with your repair? Yes No
2. Do you perform primary fascial closure with your repair? Yes No
3. Do you perform a transversus abdominis release with your repair? Yes No

I don’t know what this is

1. Do you consider yourself an expert when it comes to ventral hernia repair Yes No
